# Determinants of male involvement in family planning services in Abia State, Southeast Nigeria

**DOI:** 10.1186/s40834-022-00182-z

**Published:** 2022-08-18

**Authors:** Chidinma Ihuoma Amuzie, Uche Ngozi Nwamoh, Andrew Ukegbu, Chukwuma David Umeokonkwo, Benedict Ndubueze Azuogu, Ugonma Okpechi Agbo, Muhammad Shakir Balogun

**Affiliations:** 1 Nigeria Field Epidemiology Training Program, Abuja, Nigeria; 2 Department of Community Medicine, Federal Medical Centre, Umuahia, Abia State Nigeria; 3 Department of Community Medicine, Alex Ekwueme Federal University Teaching Hospital, Abakaliki, Ebonyi State Nigeria; 4 Abia State Ministry of Health, Umuahia, Abia State Nigeria

**Keywords:** Determinants, Family planning services, Male involvement, Nigeria

## Abstract

**Background:**

Male involvement in family planning (FP) remains low in male-dominant communities. Family planning contributes to the regulation of fertility and population growth in Nigeria. Increasing male involvement in family planning services is crucial in reducing maternal morbidity and mortality in patriarchal societies such as Nigeria. This study identified the determinants of male involvement in family planning services in Abia State, Nigeria.

**Methods:**

This was a cross-sectional study conducted in twelve communities of Abia State, Nigeria. A total of 588 married men who met the eligibility criteria were recruited using a multistage sampling technique. An interviewer-administered semi-structured questionnaire was used to collect data on the variables. Univariate, bivariate and multivariate analysis was done. The level of significance was set at 5%.

**Results:**

The overall level of active male involvement in family planning services was 55.1% (95% CI:51.0–59.2%). The mean age of the respondents was 42.4 ± 8.0 years. Access to television (aOR = 1.58, 95% CI: 1.05–2.39), spouse employment status (aOR = 2.02, 95% CI: 1.33–2.06), joint decision-making (aOR = 1.66, 95% CI: 1.05–2.62), and accompanying spouse to the FP clinic (aOR = 3.15, 95% CI: 2.16–4.62) were determinants of active male involvement.

**Conclusion:**

At least, one out of every two men was actively involved in family planning services. This was determined by access to television, employment status of spouse, joint decision-making, and accompanying spouse to the FP clinic. There is a need to focus on the identified factors in order to further improve the active involvement of men in FP services.

**Supplementary Information:**

The online version contains supplementary material available at 10.1186/s40834-022-00182-z.

## Background

Family planning (FP) programmes have centered primarily on women. However, with a focus on gender equity for optimal health, there is a shift to engage men in supporting and using FP services [1]. Men, as the decision-makers in most African families, have an important role to play towards the utilization of FP methods, which is an efficacious intervention recommended and approved by the World Health Organization (WHO) as well as the Ministry of Health (MoH) in most countries [2]. Family planning refers to a conscious effort by a couple to limit or space the number of children they want to have through the use of contraceptive methods. Benefits of family planning include reduced maternal and infant mortality, sustainable development through population control, and enhanced women’s participation in the workforce [3].

Developing countries make up about 85% of the global population and account for 99% of all maternal mortality cases [4]. According to the 2018 National Demographic Health Survey (NDHS), the maternal mortality ratio (MMR) was 512 deaths/100,000 live births [5], and Nigeria accounts for approximately one-fifth of maternal deaths globally [6]. Additionally, the lifetime risk of maternal death in Nigeria is 0.029 (1 in 34) [5], compared to 1 in 4900 in most developed countries [6]. Low level of male involvement in reproductive health practices is one of the drivers of high maternal morbidity and mortality. This has reduced the impact of family planning interventions and intertwines with unregulated fertility that hinders economic development and creates a political imbalance in a country [7, 8].

Globally, there is a growing rise in the recognition of the benefits of involving men in family planning services [9]. It is known from research that gender dominance, particularly men’s disapproval of family planning, has an impact on the subdued prevalence of contraceptive use in sub-Saharan Africa [10]. A study done in Bangladesh documented a 40% male involvement rate [4], and a similar study carried out in Western Nigeria documented 39.6% [11]. This shows that male involvement remains low despite ongoing efforts. The effect of male dominance on the decision-making process heightens the poor indices of reproductive health, as documented in a study in Nigeria where 62% of women had their husbands as their decision-makers and only 6% of currently married women at the time of the survey made decisions for themselves [5]. Male involvement in SRH (Sexual and Reproductive Health) is an integrated approach engaging men as clients, partners, and agents of positive change in reproductive health issues [12].

Access to the media, television, and radio, spouse employment status, and average monthly income have all been identified as positive correlates of male involvement in studies [11, 13–16]. However, there is a paucity of data on the factors affecting the male involvement of men in family planning services in our study location. There is a need to generate data to inform decisions taken by policymakers in designing family planning programmes. Therefore, we aimed in this study to identify the determinants of male involvement in family planning services in Abia State.

## Methods

### Study design and setting

This was a community-based household cross-sectional study that was conducted from September to December 2019 in 12 communities of Abia State in southeastern Nigeria. The State had an estimated population of 3,901,620 in 2018 projected from the 2006 national population census with an annual growth rate of 2.7% [17]. Geopolitically, Abia State is divided into three Senatorial Zones—Abia North, Abia South, and Abia Central—with 17 Local Government Areas (LGAs) and has 291 political wards. Igbo language with varying dialects, and English are the major languages for communication. Abia State is inhabited mostly by the Igbo ethnic group, who are predominantly Christians with a few people who practice traditional religion. The Catholic doctrine forbids the use of modern family planning methods.

There are 517 public primary healthcare centres, 17 public secondary healthcare facilities, and two public tertiary healthcare centres. Family planning services are available across all health facilities and can be assessed at all levels of health facilities in the state, including chemist stores and private health facilities. There are no known existing taboos against family planning use in the state.

### Sample size determination

Estimation of sample size was done using the sample size formula for cross-sectional studies [18]. A minimum sample size of 616 was determined at a confidence level of 95%, a design effect of 1.5 with a margin error of 5%. This was based on the proportion of male involvement in reproductive services (30.9%) in a previous study [11]. A non-response rate of 20% was assumed.

### Study population and sampling strategy

The study population included men in a marital/cohabiting relationship with a spouse or partner in the selected communities. This category of men is believed to have had some experiences relating to reproductive health issues in marriage and/or fatherhood. Participants were included in the study if they met the eligibility criteria of being in the age group (15–59 years) as defined by NDHS 5, in a marital or cohabiting relationship, and living in the study area 6 months prior to the study. However, those with debilitating illnesses such as cerebrovascular diseases that could interfere with communication were excluded. A total of 616 men were recruited using the multistage sampling technique. **Stage one:** Six LGAs were selected using the balloting technique. They included Aba North, Umuahia North, Ohafia, Ugwunagbo, Bende, and Ikwuano LGAs. **Stage two:** In each LGA selected, the list of communities was obtained and they served as clusters. In each of the LGAs, two clusters were selected using a simple random sampling technique. **Stage three:** All the households in each cluster were enumerated. The respondents were proportionally allocated based on the number of households in each cluster. We used computer-generated random numbers to select the households. In each of the household visited, only one eligible respondent was selected. In households with more than one eligible respondent, simple random sampling was used to select only one of them. The process was continued until the required sample size was attained in each cluster.

### Study tool and data collection process

A pre-tested interviewer-administered semi-structured questionnaire (Additional file 1) with open- and closed-ended questions was used to collect information from the participants by trained research assistants. The questionnaire was adapted from previous studies [11, 19]. The questionnaire was assessed for content and face validity and the Cronbach’s alpha index was 0.71. The Igbo translated version which was translated back to English to ensure that the original meaning was maintained, was also available for use. The questionnaire used for this study has three sections. "Background" SSection 1 addressed sociodemographic and socio-economic variables such as age, marriage type, educational status, occupational status, religion, and denomination, income, access to mass media, number of living children, educational status of spouse, and employment status of spouse. Section 2 included socio-cultural variables such as decision-maker on FP issues, accompanying spouse to FP clinic, and community and family support for accompanying spouse to FP clinic. Section 3 contained composite questions to measure the level of male involvement in family planning services. These included; Are you currently using any family planning method (s)? Have you ever discussed FP with your spouse/partner? Are you aware of any male FP method (s)? Have you ever attended any FP clinic? Have you ever discussed FP with a friend? And would you recommend FP to a friend?

There was no compensation for the respondents participating in this survey. Revisits was done up to three times to potential participants.

### Quality control and data management

The research assistants were properly trained to ensure accuracy in data collection**.** The questionnaire was pre-tested to detect and correct possible errors and identify any ambiguities before the initiation of the study using sixty (60) respondents (10% of the study sample size) in Old Umuahia (Umuahia South LGA) which was not selected for the study,

### Measurement of variables

The dependent variable was the level of male involvement in family planning services. It was created as a composite variable comprising six (6) questions covering respondents’ FP practices and FP perceptions. The responses were dichotomized (Yes/No), with a score of ‘No’ = 0 and ‘Yes’ = 1. This gave a maximal score of six (6) and a minimum score of zero (0). A total score of 0 was classified as ‘None involvement’, while a score of 1–3 was classified as ‘passive involvement’ and a score of 4–6 was classified as ‘active involvement’. For the logistic regression, a score of 0–3 was recoded as ‘passive involvement’. Additionally, active involvement was coded as ‘1’ and passive involvement coded as ‘0’ for the binary logistic regression analysis. The independent variables included age, educational status, occupational status, average monthly income, number of living children, educational status/employment status of spouse, decision-maker on FP issues, accompanying spouse to FP clinic, community and family support on accompanying spouse to FP clinic.

### Statistical analysis

Data coding, entry, cleaning, and analysis was done using IBM SPSS statistics for Windows, version 20.0. We performed univariate analysis and determined the association between the independent variables and level of male involvement in family planning services using the binary logistic regression. The variables were dichotomized for ease of data analysis and interpretation. *P* values < 0.05 and 95% confidence interval excluding the null values were considered significant. Multivariable logistic regression analysis was done to identify the significant predictors of men’s involvement in family planning services. Factors that fitted into the regression model, were those with *P* values < 0.2 at the level of bivariate analysis. Adjusted odds ratios with 95% confidence intervals were estimated and the analysis was done based on a significance level of 5%. Appropriate charts and tables were used to display the results.

## Results

### Social-demographic characteristics of the study participants

A total of 588 respondents participated in the study with a response rate was 95.5%. The mean age of the study respondents was 42.2 ± 8.0 years. Respondents were almost distributed similarly between the 35–44 years age group (41.0%) and those aged over 45 years (41.2%). Two hundred and forty-one (41.0%) had secondary education with the majority (93.2%) of them in a monogamous relationship. The majority of the respondents (55.1%) had 3–4 living children. Five hundred and seventy-three (97.4%) were Christians with more than 40% belonging to the Pentecostal denomination. Close to one-third of the respondents (31.3%) were traders and 88.9% of them had resided in their abode for more than 2 years (Table 1**).**

**Table 1:** Sociodemographic characteristics of respondents

Variable	Frequency (n)	Percentage (%)
**Age**		
25–34	105	17.9
35–44	241	41.0
≥ 45	242	41.1
Total	588	100
**Mean (±SD)**	**42.4 (±8.0)**	
**Education Status**		
No formal education	24	4.1
Primary	99	16.8
Secondary	241	41.0
Tertiary	224	38.1
**Marriage/relationship type**		
Monogamous	548	93.2
Polygamous	22	3.7
Cohabitation	18	3.1
**Current number of living children**		
None	19	3.2
1–2	129	22.0
3–4	324	55.1
≥ 4	116	19.7
**Median (IQR)**	**2.0 (2–4)**	
**Religion**		
Christianity	573	97.4
Traditional	15	2.6
**Denomination**^a^		
Catholic	131	22.9
Orthodox	188	32.8
Pentecostal	243	42.4
Others	11	1.9
**Duration at the present residence in the community**		
6 months	8	1.4
> 6–12 months	13	2.2
> 12 months-2 years	44	7.5
> 2 years	523	88.9
**Occupation status**		
Professional	28	4.8
Trader	184	31.3
Civil servant	146	24.8
Skilled manual labour	47	8.0
Artisan	71	12.1
Farming	81	13.8
No occupation	31	5.3

*IQR* Interquartile Range

^a^ *n* = 573

### Proportion of agreed responses on male involvement in family planning services by indicators

A large majority of men (84.2%) had discussed FP with their spouses in the past 6 months prior to the study. The majority (70.4%) were aware of male-focused FP methods. Only 57.3% were currently using a FP method and 64.8% had discussed FP with their friends. In contrast, less than half of the men (49.3%) had ever attended a FP clinic and recommended FP to their friends (48.5%) in the past 6 months prior to the study. **(**Table 2**).**

**Table 2:** Proportion of agreed responses on male involvement in family planning services by indicators (*N* = 588)

Variable	Yes (%)
Aware of any male-focused FP method	414 (70.4)
Currently on any family planning methods	337 (57.3)
Ever discussed FP issues with your spouse/partner	495 (84.2)
Ever attended any FP clinic	290 (49.3)
Ever discussed FP with friend	381 (64.8)
Ever reccommended FP to a friend	285 (48.5)

Multiple responses were allowed

### Socio-economic/cultural characteristics of the respondents

Two hundred and forty-one (43.3%) were in the ≥₦60,000 monthly income category. The median income was ₦50,000 (IQR: ₦30,000–₦50,000). The majority of the respondents had access to - newspapers (66.3%), radio (88.3%) and television (68.2%). Two hundred and fifty-six of the respondents’ spouses (43.6%) had a minimum of tertiary education and the majority (62.9%) of them were employed. The majority of the respondents (78.6%) made joint-decisions with their spouses on FP issues. However, close to half (49.8%) of them agreed to accompany their spouse to the FP clinic. The majority of the respondents (92.0%) agreed that FP was not solely a woman’s responsibility and more than 75% of the respondents believed that FP was supported by family members and the community **(**Table 3**).**

**Table 3:** Socio-economic/cultural characteristics of the respondents

Variables	Frequency (n)	Percentage (%)
**Monthly income**^**a**^ **(Naira ₦)**		
< ₦30,000	149	26.8
≥ ₦30,000-59,999	167	30.0
> ₦60,000	241	43.2
**Median (IQR)**	**₦50,000 (₦30,000–₦50,000)**	
**Access to Newspapers**		
Yes	390	66.3
No	198	33.7
**Access to radio**		
Yes	519	88.3
No	69	11.7
**Access to television**		
Yes	401	68.2
No	187	31.8
**Educational status of spouse**		
None	34	5.8
Primary	62	10.5
Secondary	236	40.1
Tertiary	256	43.6
**Employment status (spouse)**		
Employed	370	62. 9
Unemployed	218	37.1
**Decision maker on FP**		
With spouse	462	78.6
Spouse only	122	20.7
Others (Relatives)	4	0.7
**Accompanies spouse to FP Clinic?**		
Yes	293	49.8
No	295	50.2
**FP is solely a woman’s responsibility**		
Yes	47	8.0
No	541	92.0
**Does your family support you to accompany your spouse to the FP clinic?**		
Yes	460	78.2
No	128	21.8
**Does your community support you to accompany your spouse to the FP clinic?**		
Yes	481	81.8
No	107	18.2

*FP* Family Planning, *IQR* Interquartile Range

^a^*n* = 557

### Factors associated with active male involvement in family planning services among the respondents

Among the respondents, 55.1% (95% CI: 51.0–59.2%) were active in FP services compared to 39.6% (95% CI: 35.6–43.7%) who were passive. However, 5.3% (95% CI: 3.6–7.4%) were not involved in any form of FP services.

The participants who had access to television were more likely to be active in family planning services compared to their counterparts. (OR = 1.70, 95%CI:1.20–2.40) Respondents whose spouses were employed were 90% more likely to be actively involved in FP compared to those whose spouses were not employed. (OR = 1.90, 95%CI:1.35–2.67) Respondents who had joint decision-making with their spouses on FP issues were also more likely to be involved actively in FP services compared to those whose spouses solely took decisions. (OR = 2.15, 95%CI:1.43–3.23) Active involvement was three-fold higher in men who agreed to accompany spouses to the FP clinic. (OR = 3.39, 95%CI:2.41–4.77).

There was a positive association with the active involvement and support of family members in accompanying spouse to the FP clinic. (OR = 1.78, 95%CI:1.20–2.64) Furthermore, those who believed that their community supported accompanying spouse to the FP clinic were 80% more likely than their counterparts to be active in male involvement (OR = 1.80, 95%CI: 1.18–2.75). (Table 4**).**

**Table 4:** Factors associated with active male involvement in family planning among the respondents (*N* = 588)

Variable	Male involvement		Total	COR (95%CI)	***P***-value
	Active (n%)	Passive (n%)			
**Age**					
< 40	133 (52.2)	122 (47.8)	255	0.82 (0.59–1.14)	0.237
≥ 40	190 (57.1)	143 (42.9)	333	1	
**Educational status**					
Tertiary	162 (54.6)	135 (45.4)	297	0.97 (0.70–1.34)	0.849
Below tertiary	161 (55.3)	130 (44.7)	291	1	
**Marriage type**					
Monogamous	312 (55.2)	254 (44.8)	566	1.23 (0.52–2.88)	0.636
Polygamous	11 (50.0)	11 (50.0)	22	1	
**Denomination**^**a**^					
Catholic	76 (55.5)	61 (44.5)	137	1.02 (0.70–1.51)	0.884
Non-Catholic	247 (54.8)	204 (45.2)	451	1	
**Current number of living children**^**┼**^					
≥ 4	164 (53.2)	144 (46.8)	308	0.87 (0.62–1.21)	0.409
1–3	148 (56.7)	113 (43.3)	261	1	
**Occupational status**^**a**^					
Skilled	139 (59.4)	95 (40.6)	234	1.33 (0.95–1.87)	0.097
Unskilled	169 (52.3)	154 (47.7)	323	1	
**Monthly income**^**a**^ **(Naira ₦)**					
≥ ₦55,000	174 (55.6)	139 (44.4)	313	1.03 (0.73–1.43)	0.874
< ₦55,000	134 (54.9)	110 (45.1)	244	1	
**Access to Newspaper**					
Yes	225 (57.7)	165 (42.3)	390	1.39 (0.99–1.96)	0.059
No	98 (49.5)	100 (50.5)	198	1	
**Access to radio**					
Yes	291 (56.1)	228 (43.9)	519	1.48 (0.89–2.44)	0.128
No	32 (46.4)	37 (53.6)	69	1	
**Access to Television**					
Yes	237 (59.1)	164 (40.9)	401	1.70 (1.20–2.40)	0.003*
No	86 (46.0)	101 (54.0)		1	
**Educational status of spouse**					
Tertiary	174 (52.4)	158 (47.6)	332	0.79 (0.57–1.10)	0.162
Below tertiary	149 (58.2)	107 (41.8)	256	1	
**Employment status (spouse)**					
Employed	225 (60.8)	145 (39.2)	370	1.90 (1.35–2.67)	< 0.001*
Unemployed	98 (45.0)	120 (55.0)	218	1	
**Decision maker on FP^**					
Jointly with spouse	273 (59.1)	189 (40.9)	462	2.15 (1.43–3.23)	< 0.001*
Spouse only	49 (40.2)	73 (59.8)	122	1	
**Escorts spouse to FP Clinic**					
Yes	204 (69.6)	89 (30.4)	293	3.39 (2.41–4.77)	< 0.001*
No	119 (40.3)	176 (59.7)	295	1	
**FP is a woman’s duty**					
Yes	23 (49.0)	24 (51.0)	47	0.77 (0.42–1.40)	0.389
No	300 (55.5)	241 (44.5)	541	1	
**Family support to accompanying your spouse to the FP clinic**					
Yes	267 (58.0)	193 (42.0)	460	1.78 (1.20–2.64)	0.004*
No	56 (43.8)	72 (56.2)	128	1	
**Community support accompany your spouse to the FP clinic?**					
Yes	277 (57.6)	204 (42.4)	481	1.80 (1.18–2.75)	0.006*
No	46 (43.0)	61 (57.0)	107	1	

*COR* Odds Ratio, *CI* Confidence Interval

Binary logistic regression

^a^*n* = 557 ┼ *n* = 569 ^*n* = 584 *p values < 0.05 are considered significant

### Predictors of active male involvement in family planning services

Male involvement in family planning services was predicted by access to television (aOR = 1.58, 95% CI: 1.05–2.39), spouse employment status (aOR = 2.02, 95% CI: 1.33–2.06), joint decision-making (aOR = 1.66, 95% CI: 1.05–2.62), and accompanying spouse to the FP clinic (aOR = 3.15, 95% CI: 2.16–4.62). **(**Table 5**)**.

**Table 5:** Predictors of active male involvement among the respondents

Variable	aOR (95%CL)	***P*** value
**Occupational status**^**a**^		
Skilled	0.98 (0.64–1.49)	0.924
Unskilled	1	
**Access to Newspaper**		
Yes	0.94 (0.62–1.41)	0.749
No	1	
**Access to radio**		
Yes	1.30 (0.73–2.32)	0.369
No	1	
**Access to Television**		
Yes	1.58 (1.05–2.39)	0.028*
No	1	
**Educational status of spouse**		
Tertiary	1.29 (0.83–1.99)	0.256
Below tertiary	1	
**Employment Status (Spouse)**		
Employed	2.02 (1.33–3.06)	0.001*
Unemployed	1	
**Decision maker on FP**		
Jointly with spouse	1.66 (1.05–2.62)	0.029*
Spouse only	1	
**Accompanies spouse to FP Clinic?**		
Yes	3.15 (2.16–4.62)	< 0.001*
No	1	
**Does your family support accompanying your spouse to the FP clinic?**		
Yes	1.26 (0.77–2.07)	0.351
No	1	
**Does your community support accompany your spouse to the FP clinic?**		
Yes	1.31 (0.78–2.21)	0.298
No	1	

*aOR* Adjusted Odds Ratio, *FP* Family Planning

Multivariable logistic regression *p values < 0.05 are considered significant

## Discussion

We conducted this study to determine the level of male involvement and its predictors in family planning services among men of Abia state, southeastern Nigeria. We found out that at least, one out of two men was active in FP services. Access to television, the spouse’s employment status, joint decision-making, and accompanying the spouse to the FP clinic were the predictors of male involvement in FP services.

The findings in this study showed that slightly more than half of the respondents were actively involved in FP services. This is in contrast to a study done in Ogun State, Nigeria, that noted an active involvement rate of 30.9% [11]. Additionally, researchers have reported lower rates of active involvement in Ghana (34.5%), Ethiopia (44%) and Bangladesh (40%) [4, 19, 20]. However, a recent study in Ethiopia reported a higher active involvement rate of 68%, while an earlier study in an urban municipality in Bangladesh noted a male involvement rate of 63.2% in FP [14, 21]. Poor involvement could be attributed to the patriarchal societies that exist in the African context, few male family planning methods and the prevailing myths and misconceptions associated with family planning use [8, 20, 22, 23]. Improving the services available for men and disseminating accurate information on the associated myths and misconceptions associated with FP services should be encouraged.

Access to television was a significant factor in determining active involvement in FP services by men. Access to the media is likely to enhance attitudes and behaviour change leading to improved male involvement in FP. Some researchers have also observed these findings in their various studies [11, 14, 24–27]. In Nigeria, the mass media play a crucial role in disseminating health information and increasing awareness about health education.^13^ This, over time, changes the attitude and behaviour of the masses to achieving optimal health [13]. Seeing FP messages on television and hearing them on the radio are associated with reported modern FP use [28]. The media plays an important role in attenuating the public perception of risks and provides a key link in the risk communication process. Efforts should be made to increase media coverage, especially in areas where they are not easily accessible.

The employment status of the spouse was a predictor in this study. This finding is consistent with the results of studies done in Ogun State and Bangladesh [11, 14]. Women who are employed are likely to be involved in decision-making [29]. Decision making is paramount in the uptake of reproductive health services. Additionally, women who are employed tend to plan their family size in such a way as to avoid hindrances to their services at their workplaces. Men should be encouraged to allow their spouses seek for jobs and women should be made to understand the benefits of getting employed.

Men who accompanied their wives to the FP clinic were more likely to use family planning services. This is consistent with a South African study which admitted that social support and joint responsibility for family planning and contraceptive use (FP/C) positively influence male participation [30]. However, the finding of a study in Osun State is at variance with this result [31]. Accompanying wife to FP clinic is likely to influence involvement in FP services because it is an outcome of spousal communication and joint decision making, which play a vital role in reproductive health issues.

Respondents who made joint decisions with their spouses or partners had an increased odds of being involved in family planning services. This is similar to findings from an earlier study conducted in Cross River State, Nigeria where the likelihood of using FP services increased when the decision was made jointly by both husband and wife [32]. This is also comparable to a study in Ethiopia which noted discussion with the spouse about FP issues to be a significant factor of male involvement [33]. A qualitative study in Malawi documented that joint decision-making in FP responsibilities is assisted by male involvement [34]. Furthermore, higher odds of male involvement were reported among men who jointly participated in decision-making with their partners [35]. Men are known to be culturally dominant and are expected to meet the sociocultural expectations and values attached to women and marriage [36, 37]. Men are beginning to accept the key messages of reproductive health services, and as such, take decisions that positively influence their involvement in FP practices.

The major strength of this study was that men were directly interviewed, instead of using their spouses as proxies. This gave the men better opportunities to express their opinions, ideas, and views more confidently. It was also a community-based study which would increase the generalizability of the study’s findings. Concurrently, the limitations of this study included: being a cross-sectional study, causal inferences cannot be conclusively made; the certainty of recall bias and social desirability bias. Additionally, there was no single index for measuring male involvement at the time of this study, this might have contributed to the variances observed with similar studies. These were, however, mitigated by assuring the respondents of their confidentiality and privacy, and an extensive literature review was done to select the suitable questions used for measuring the dependent variable.

## Conclusion

The prevalence of active involvement in FP services was 55.1%. This was influenced by access to television, employment status of spouse, joint decision-making and accompanying spouse to the FP clinic. We recommend FP sensitization campaigns targeting men to encourage their participation in FP services. There is a need to improve the existing family planning programmes with a focus on the identified factors in order to enhance the active involvement of men in FP services.

## Supplementary Information

**Additional file 1.**

## Data Availability

The dataset analyzed in this study are available from the corresponding author on reasonable request.

## Abbreviations

aOR: Adjusted Odds Ratio
CPR: Contraceptive Prevalence Rate
FP/C: Family planning/contraceptive use
FP: Family Planning
ICPD: International Conference on Population and Development
IQR: Interquartile range
LGA: Local government area
MMR: Maternal Mortality Ratio
NDHS: Nigeria Demographic Health Survey
NFELTP: Nigeria Field Epidemiology and Laboratory Training Program
OR: Odds ratio
SRH: Sexual and Reproductive Health
TFR: Total Fertility Rate
WHO: World Health Organization
