# Supplementary material for: Determinants of male involvement in family planning services in Abia State, Southeast Nigeria
Source: Contracept Reprod Med. 2022 Aug 18;7:15. doi: 10.1186/s40834-022-00182-z (PMC9386938; doi:10.1186/s40834-022-00182-z)
Supplement: Supplementary file 1 — Additional file 1. [file 40834_2022_182_MOESM1_ESM.docx]

**DETERMINANTS OF MALE INVOLVEMENT IN FAMILY PLANNING SERVICES IN ABIA STATE, SOUTHEAST NIGERIA**

CODE……………………………

Dear respondent,

We are a team of researchers conducting research on “**DETERMINANTS OF MALE INVOLVEMENT IN FAMILY PLANNING SERVICES IN ABIA STATE, SOUTHEAST NIGERIA**”. Your opinion, experiences and responses on this subject are very important to us and will be treated as anonymous and your information will be handled confidentially.

This interview is voluntary and you are free to opt out anytime you so desire. So, feel free to give us your frank and honest opinion. If you will not object, we may have need to use an audio recorder to record what you are saying, so that we do not forget anything you said and will also write down some of your statements.

I hope you understood everything I said. Do we have your permission to go ahead?

YES……………… NO……………….

Thank you.

**SECTION 1: SOCIO-DEMOGRAPHIC/ SOCIO ECONOMIC VARIABLE**

1. Age as at last birthday (in years)? …………
2. Level of education completed: No formal education [ ] Primary School [ ] Secondary School [ ] Tertiary[ ]
3. Type of marriage: Monogamous [ ] Polygamous [ ] Cohabitation [ ] Others…….
4. Are you living with your Spouse? Yes [ ] No [ ]
5. What is your Religion? Christianity [ ] Islam [ ] Traditional [ ] Others……………..
6. What is your denomination? Catholic [ ] Orthodox [ ] Pentecostal [ ]
7. How long have you lived here?...................................
8. What is your occupational status? Professional [ ] Trader [ ] Civil Service [ ]

Skilled manual labour [ ] Artisan [ ] Farming [ ] Others (specify)……………..

1. Average monthly income (Naira)…………………………….
2. Daily access to media YES NO

Do you read newspaper regularly? [ ] [ ]

Do you listen to radio regularly? [ ] [ ]

Do you watch television regularly? [ ] [ ]

1. How many living children do you have?: ……………
2. Educational status of wife : No formal education [ ] Primary School [ ]

Secondary School [ ] Graduate and above [ ]

1. What is your wife’s employment status? Employed [ ] Unemployed [ ]

**SECTION 2 : SOCIOCULTURAL VARIABLES**

1. Who is the decision maker on family planning issues in your home? Jointly with spouse [ ] Wife [ ] Others (specify) …………………….
2. Do you usually accompany your wife to the family planning clinic? Yes [ ] No [ ]
3. Do you think your community considers it acceptable for a man to accompany his wife/ partner to family planning clinic? Yes [ ] No [ ]
4. Is family planning solely a woman’s responsibility? Yes [ ] No [ ]
5. Do you think your family and friends consider it acceptable for a man to accompany his wife/ partner to family planning clinic? Yes [ ] No [ ]

**SECTION 3 : MALE INVOLVEMENT IN FAMILY PLANNING SERVICES**

1. Are you aware of any male family planning method? Yes [ ] No [ ]
2. Are you currently using any family planning method(s)? Yes [ ] No [ ]
3. Have you ever discussed family planning with your spouse/partner? Yes [ ] No [ ]

1. Have you ever attended any family planning clinic? Yes [ ] No [ ]
2. Have ever discussed family planning with your friend? Yes [ ] No [ ]
3. Have you ever recommended family planning to a friend? Yes [ ] No [ ]

**THANK YOU FOR YOUR TIME**
